# The state of emergency medicine in Greece: at critical momentum

**DOI:** 10.1186/s12245-024-00622-4

**Published:** 2024-04-03

**Authors:** Dimitrios Tsiftsis, Andrew Ulrich, George Notas, Anna Patrikakou, Eleanor Reid

**Affiliations:** 1 Emergency Department, Nikaia General Hospital, D. Mantouvalou 3, Nikaia, 18454 Greece; 2 Department of Emergency Medicine, Yale University School of Medicine, 464 Congress Avenue, Suite 260, New Haven, CT USA; 3 University of Crete School of Medicine and University Hospital of Heraklion, 13 Andrea Kalokairinou Str., Heraklion, 71500 Greece; 4 2nd Regional Health Authority of Piraeus and the Aegean, Thivon 196, Agios Ioannis Rentis, 18233 Greece

**Keywords:** Emergency medicine development, Greece

## Abstract

Greece is a parliamentary republic in southeastern Europe populated by over 10 million permanent residents: 9 million reside on the mainland, with almost 4 million in the greater Athens area. The remaining 1 million populate the over 1200 Greek islands. In addition, more than 160,000 asylum-seekers reached Greece in 2022, and more than 25 million tourists have visited Greece in the last two years. Modern Greek Emergency Medicine (EM) is now in its 4^th^ decade. The Greek government has focused the last few years on enhancing the quality of emergency services provided in public hospitals. Emergency Departments (EDs) are being modernized, undergraduate medical education gradually incorporates EM, and a specialty training program in emergency nursing has been established. However, the late recognition of the critical importance of EM as a specialty in Greece has resulted in the subsequent need to create three alternative pathways to EM, none of which are direct from residency. The first is a 24-month Emergency Medicine fellowship after completing a residency in another specialty and then passing the national exam. The second is for physicians who have worked in a public hospital ED (*Gr: Ethniko Systima Ygeias* (ESY) ESY for at least three years and successfully passed the national exam. The third, which no longer exists, is a ‘grandfather’ pathway for those physicians who worked in an ESY ED for five years prior to the creation of the fellowship training program. As a result, there is a critical shortage of EM-trained physicians, resulting in most care being provided by physicians without formal training in EM. This is further confounded by the country’s challenging geography, with frequent air transfers from the islands to mainland hospitals. Creating an EM Residency training program is a critical next step to overcoming many of the challenges facing EM provision in Greece today: it would address the shortage of EM-trained providers, decrease the need for costly ground and air transfers, and improve the quality of emergency care throughout Greece.

## Background

The history of medicine begins in Greece, with Asclepius and Hippocrates describing a holistic philosophy and art of medicine emphasizing the intertwined relationship of physical and mental health, and the physician’s role in upholding ethical standards. This is represented in the Hippocratic Oath, the earliest version of which dates to AD 275, yet its principles continue to guide medical practice 2500 years later: first, do no harm [1]. Emergency medicine also had a central role in medicine in ancient Greece: the Spartan military had dedicated medical personnel for battlefield emergencies, familiar with the principles of wound care [2, 3]. In comparison, the history of modern emergency care in Greece and the state of Greek Emergency Medicine (EM) today are far less well-documented.

Whilst emergency care exists in any healthcare system by default, Emergency Departments (EDs)have existed in modern Greece only recently. Developing high-quality emergency care was not always a priority of the Greek government, having only begun in 2019. However, as the benefits of EM have become more evident at a national level, including improved outcomes for patients with acute illness, improved organization of EDs, the efforts of a tireless group of stakeholders are credited with pushing Greek EM forward.

This report focuses on EM development achievements and challenges in Greece and the steps we need to undertake as we take advantage of the current critical momentum.

## Current landscape

### Geography

**Fig. 1:**
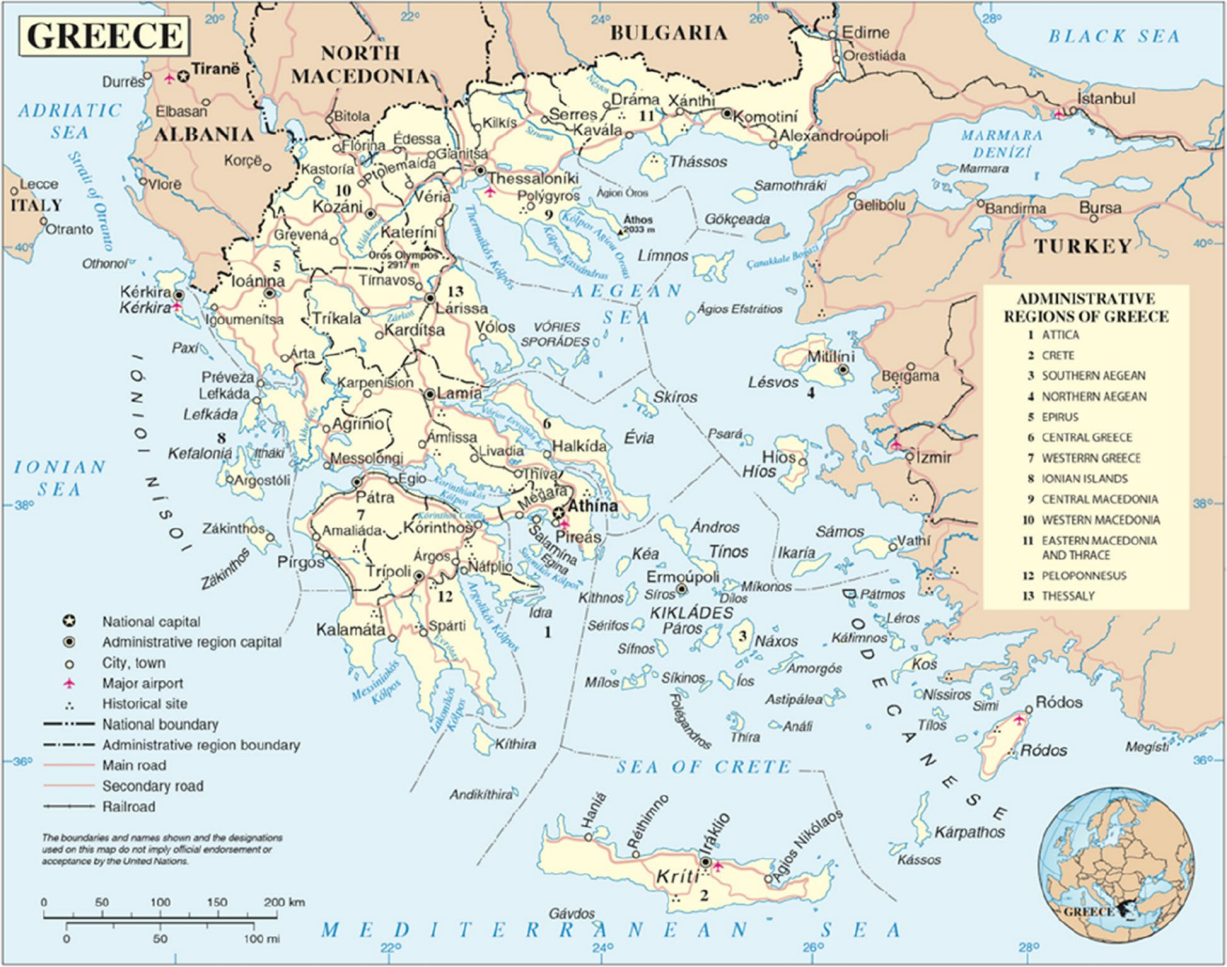
Map of Greece [1]

Greece is a parliamentary republic located in southeast Europe and is a member of the European Union. Greece has a total surface area of 131,957 km^2^, divided between the mainland and more than 1200 Greek islands (Fig. 1). Greece has a population of over 10 million permanent residents; 9 million reside on the mainland, with nearly 4 million in the greater Athens area. The remaining 1 million populate the sparsely inhabited islands: only 10 islands have a population of 30,000 or more, while the vast majority have a population between 1,000 and 20,000 permanent residents [4].

### The national health system

In 1983, Greece passed a law establishing a national health system (*Gr: Ethniko Systima Ygeias* (ESY)), universal coverage of the insured population, and equal access to health services, with the government fully responsible for providing all services to the population (Law 1397/83). Primary care was established in health centers staffed by general practitioners (GPs). Specialist services were made available through hospital outpatient departments, state-run medical offices, and private practices [5]. A Central Health Council (Gr: *Kentriko Symvoulio Ygeias* (KESY)) was established as an advisory role to the Ministry of Health on policy and research issues. ESY doctors, nurses, and other personnel are required to work exclusively at the ESY, with identical salaries for all medical specialties (compensations are added for years of service, marital status, administrative responsibilities, etc.). The 2010s austerity period was challenging for the ESY, leading to the significant exodus of well-trained personnel towards the private sector, or other EU countries.

Despite a socialized system, the average Greek citizen reports spending 600€ per year on medical care, which negatively affects healthcare-seeking behavior. For comparison, the minimum wage in Greece is currently set at 780€/month [6].

### Prehospital emergency services

**Fig. 2:**
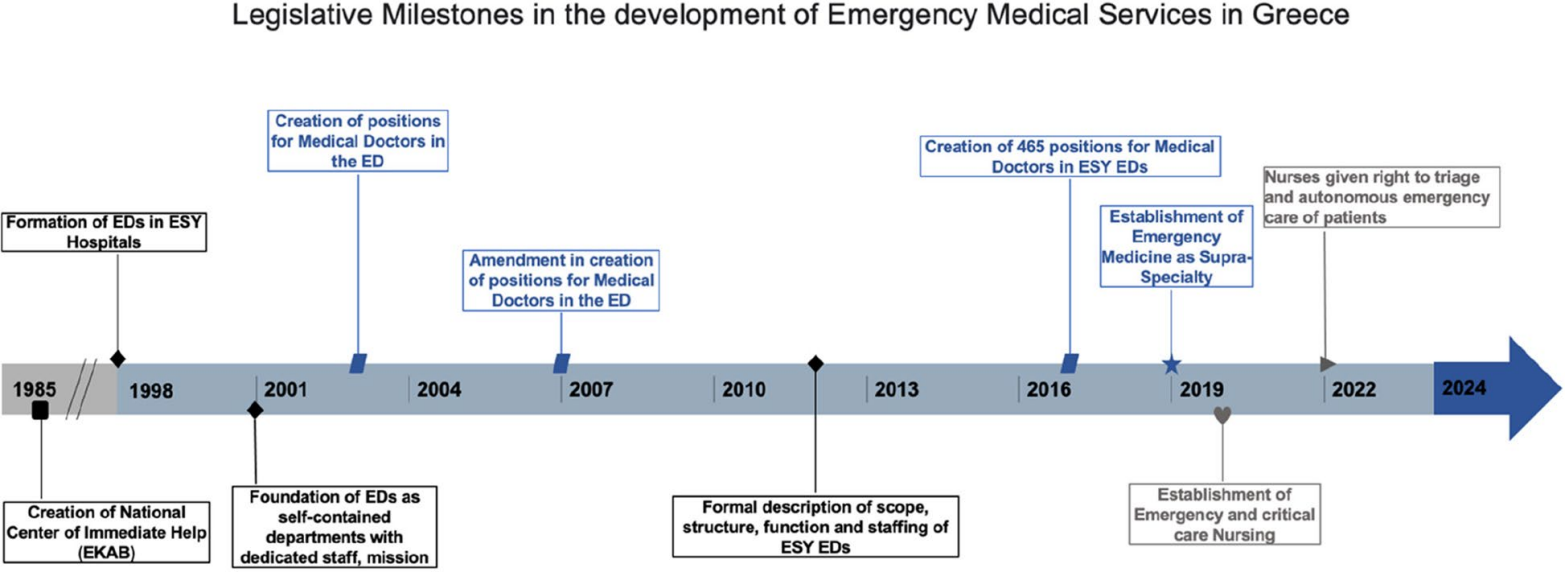
Timeline of legislative milestones in the development of emergency medical services in Greece

Pre-hospital emergency care is provided through ESY and the National Center of Immediate Help (Gr: *Ethniko Kentro Amesis Boithias* (EKAB)), which was established in 1985 (Fig. 2). EKAB is responsible for providing pre-hospital emergency care to all citizens and transportation to healthcare facilities. It also provides for continuing training of doctors, nurses, and other healthcare personnel in pre-hospital care. EKAB is headquartered in Athens, with branches in most regions, serving 500,000 patients annually. EKAB’s Command and Coordination Centre is the first contact point for emergency care, receiving all emergency calls through two nationwide three-digit call numbers (166 or 112). It also selects and mobilizes the most appropriate response, guides ambulance crews in providing specialized life support, and coordinates with hospital EDs. Pre-hospital care in Greece follows the Anglo-American model of providing transport to the ED, or ‘scoop and run’ philosophy. Ambulance crews in Greece include two levels of providers: post-residency physicians who have completed a 12-month course in pre-hospital EM and paramedics certified after a 2.5-year post-high school training program.

### In-hospital emergency care

In-hospital emergency care is provided through ESY hospitals’ EDs (*Gr: Tmimata Epeigonton Peristatikon* (TEP)), which were established in every ESY hospital in Greece in 1998 (Fig. 2). The EDs were to be run by a team of physicians from different medical specialties already appointed in the hospitals’ other medical departments, who would rotate for 12 months in the ED. These doctors were tasked with covering their specialty’s emergencies only. From time to time, a small number of permanent ED positions were provided in selected large hospital EDs. In some rare instances, doctors with a particular interest in EM would choose to spend more time in the ED, but their appointed positions remained within the department of their main specialty. This type of organization has since been modified to the current system, which is presented in full below. What remains unchanged is an unconventional system of some EDs operating not on a 24/7 basis but, depending on the size of the city and the number of hospitals, from once every four days to every other day.

### Current state of Emergency Medicine in Greece

In 2017 the Greek Ministry of Health opened 465 permanent positions for doctors in EDs, thus increasing the total number of available permanent ED positions to 580. These positions were still only a fraction of what would be needed, but it was a start. Since the country did not have EM training in any form, the openings were for other medical specialties, with an advantage given to Critical Care physicians. Of these positions, 324 out of 465 were filled, resulting in EDs having their own medical personnel for the first time. Several of these positions were for medical directors, enabling for the development of EM in Greece.

However, understaffed hospital departments claimed the newly hired specialists which matched their needs and pushed administrators to move these new hires out of the ED, to the clinics to meet existing needs. Some hospital administrations chose this as there was no prior culture of EM. When the COVID-19 pandemic hit, focus was shifted to hospitalized COVID-19 patients. By November 2022, 78/324 doctors initially hired in an ED were providing their services in other hospital departments. This use of ED doctors to cover system gaps continues to this day. Due to low staffing, the allocation of ED doctors to other departments, or the exit of ED personnel without timely replacement has resulted in EDs losing the minimum number of active members to form a critical mass capable of advancing their ED.

#### The Hellenic Society for Emergency Medicine (HeSEM) [7]

The Hellenic Society for Emergency Medicine (HeSEM) was founded in 2002. For years, physicians in EKAB, ESY, and academics advocated for a formal emergency medicine training program through HeSEM. All existing emergency medicine training programs today derive from the constant efforts of these pioneers. In the 2010s, the HeSEM released several positional statements concerning ED staffing, EM board examinations, and the rights and responsibilities of physicians working in EDs as emergency physicians or with their primary specialty. The Ministry of Health has adopted some of these, while more recent position papers are in evaluation by the Central Health Council (KESY). Currently, the HeSEM has initiated an endeavor to harmonize the training and functions of this diverse field by providing a unified set of congresses, seminars, webinars, and recommendations, as well as the first National Triage Guidelines (*Gr: Ethniko Systima Dialogis*), which are currently in final draft.

### Clinical pathways to Emergency Medicine training in Greece: a “supra-specialty”

There are three pathways for physicians to receive the National Diploma of Emergency Medicine in Greece, none of which are direct routes from medical school. The first is through a supra-specialty program. EM was initiated as a supra-specialty in 2019 after persistent pressure from the HeSEM. Since then, doctors having graduated from residency training programs with the specialties of General Medicine, Anesthesiology, Internal Medicine, Pediatrics, Surgery, Orthopedics, Thoracic Surgery, and Cardiology can start a 24-month, post-residency fellowship in EM, followed by a national exam, leading to the “Title of the Supra-Specialty of Emergency Medicine (*Gr: Titlos eksideikeysis stin epeigousa Iatriki*)). In 2020, the first two EM fellowship training programs began in Athens and Heraklion, offering 36 training posts. Today, nationally recognized fellowship programs are also offered in Thessaloniki and Patras. To date, 13 physicians (11 female and 2 male) have completed the fellowship, with a 100% success rate.

The second pathway involves physicians working in an ESY ED for at least three years after their primary specialty. These doctors may sit for the national exam for EM. The successful pass rate of non-fellowship-trained physicians is constantly rising [8]. In 2021, the first ESY-ED EM physicians were certified through this pathway. The third pathway was a “grandfathering phase” and no longer exists. It allowed physicians working in an ESY ED for five years before the initiation of the supra-specialty, or for two years in an ED director position, to be recognized as EM physicians without sitting for the national exam. Today a total of 176 physicians hold the title of the Supra-Specialty of Emergency Medicine [9].

### The role of Greek medical schools in Emergency Medicine development

Prior to 2018, EM was rarely included in Greek undergraduate medical education. The University of Crete was an early pioneer, offering medical students EM as a preclinical course starting in 1995 and as a clinical rotation in 2012. Today, the discipline of EM has been established in most medical schools, which offer pre-clinical courses, clinical rotations, and post-graduate master’s programs. Most academics that teach EM in medical schools are affiliated with EDs in University hospitals, which are incorporated into the functions of ESY. Their academic institution regulates their teaching and research, as well as their salary and obligations through the Ministry of Education, while their work in the hospital is regulated by the Ministry of Health and is compensated based on their academic position. Since none of them had formal training in EM, they received the supra specialty via grandfathering.

The number of faculty in the field of Emergency Medicine is increasing. Nationwide, seven academicians are currently at different stages of their careers. Thus, the depth of teaching and Emergency Medicine research base is expanding.

### Infrastructure modernization

**Fig. 3:**
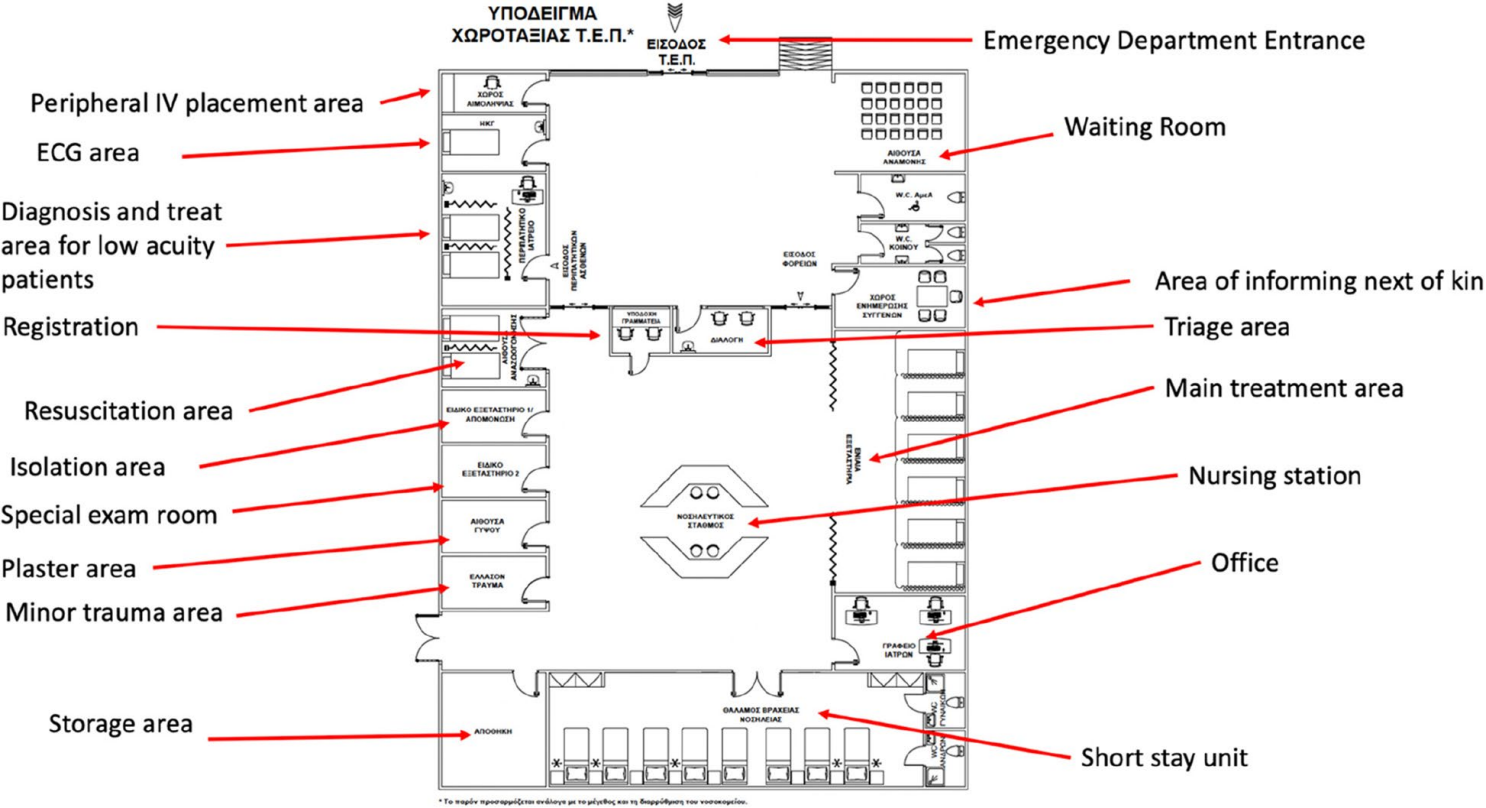
Greek Ministry of Health indicative blueprint for modernizing emergency departments [1]

Most EDs in Greece were created in the 1980s and the 1990s in existing hospitals. In some cases, outpatient departments were repurposed to become EDs, in others, modifications were made to existing buildings. In the absence of EM as a distinct discipline, having multiple, separate per-specialty emergency rooms within an ED was the norm. As more ED directors have been appointed, and with this a paradigm shift in thinking surrounding EM, there is currently an effort to abandon this model. In 2022, the Greek government announced a program of rebuilding most ESY EDs and designed blueprints for modern, non-specialty-based EDs (Fig. 3).

### Current challenges

The late recognition of the critical importance of EM as a discipline in Greece has resulted in a series of complexities. First, the provision of EM in geographically remote places: there is an extreme shortage of trained EM providers in Greece, resulting in ESY care provided mainly by physicians without formal training in EM. The country’s challenging geography further confounds this. In rural locations and on many smaller islands, the only medical provider might be a recent medical graduate posted for obligatory rural service by the Greek government (Gr: *Agrotiko*) or a General Practice health center. Mainly in the islands, acute conditions are transported by air. Though the flight time to an adequately organized ESY hospital may be less than an hour, there may be delays up to 12 h, depending on weather conditions. In total, more than 1200 patient airlifts have been performed in 2023 by the Greek Armed Forces (Gr: *Aerodiakomides – Polemiki Aeroporia*) at a not insignificant cost, borne by the Greek government [10].

A second complexity is the lack of dedicated ED personnel and in particular, those with EM training. As stated earlier, starting in 2019, some physicians have been permanently posted in the EDs of these hospitals, most of them have no formal training in EM. Even in the most prominent and busiest EDs in Athens, there are not EM physicians to staff the EDs fully. As a result, attendings and residents from various specialties assist in rotating shifts. According to data from the HeSEM, in early 2022, fewer than 250 physicians nationwide held a permanent ED position, with 160 of these in the greater Athens area. Of these, only 24% (60) were certified EM Physicians.

Third, Greece has large population changes and seasonal variations. Due to its geographic location, people fleeing violence from the Middle East, South and Central Asia, and recently, Ukraine, use Greece as a gateway to Europe. Greece remains one of the most accessible passageways for refugees and immigrants to enter Europe. More than 160,000 asylum-seekers reached Greece in 2022 alone [11]. Greece currently hosts approximately 120,000 refugees, of which 20,000 are on the islands, but unofficial numbers are much higher.

Greece remains an important tourist destination all year long, with more than 25 million visitors per year. Islands with healthcare services designed for a population of a few hundred face significant challenges when the population swells during the summer. Refugees, tourists and Greek nationals are all afforded the same access to emergency care.

## Discussion

Progress in Greek EM development over the past forty years has been significant. However, EM development is lagging compared to other EU countries. Much remains to be done, and many challenges to overcome in order to develop the discipline further and meet the needs of the millions of Greek patients, visitors, and refugees.

First, doctors and nurses working in EDs must have a clear and distinct role within the ESY and be recognized as practicing EM. The rights and obligations of emergency health professionals need to be regulated, with clear parameters for scope of practice. EDs must be self-sufficient and open for emergency care 24 h a day. The current staffing of most Greek EDs by various non-EM specialists must be redesigned by providing a well-paved pathway for continuing medical education to those ED ESY doctors that will better prepare them for the complete skillset of EM.

Second, national clinical guidelines, and policies must be established. A national triage system could be a significant starting point to standardize processes, policies, and acuity terminology across all EDs in Greece. Key performance indicators (KPIs) need to be set to identify systemic dysfunctions or best clinical practices and establish policy and research priorities.

Finally, it is time for the development of an Emergency Medicine Residency. To this end, HeSEM, in collaboration with international partners (EUSEM, ACEP, IFEM), has presented an EM residency program in accordance with international standards that are appropriate for and tailored to the Greek context, that is currently under evaluation at KESY.

From a health economics standpoint, investing in EM will likely have a significant cost-saving effect [12]. Employing EDs with adequately trained EM physicians will decrease staffing needs for the coverage of many specialty-related emergency cases and reduce the number of costly trans-hospital transfers, while allowing specialists currently covering the ED to focus on their primary specialty.

In Greece, a confluence of factors has come together to create a state of critical momentum in the development of Emergency Medicine. Most important is perhaps buy-in from the Ministry of Health with downstream effects of creation of an Emergency Medicine supra-specialty for physicians and specialty emergency training for nurses, rebuilding all EDs and creating permanent staff positions in EDs. Meaningful work is being done as these words are written, EDs are being restructured, and the number of EM supra-specialists is increasing. We have a critical mass of trained staff working in EDs, and need the ongoing support of the government as we look to next steps to further develop EM in Greece: by creating Greece’s first Emergency Medicine Residency training program. With appreciation of the efforts of those countries that have worked to establish EM, Greece will build upon the existing knowledge and science to create a culture and tradition of EM specific and unique to the Greek context.

Hippocrates is credited with the following Aphorism, which was written somewhere between the late fifth and middle fourth century BC and powerfully resounds with the state of Emergency Medicine in Greece today:

*Ὁ βίος βραχύς, ἡ δὲ τὲχνη μακρὴ, ὁ δὲ καιρὸς ὀξὺς, ἡ δὲ πεῖρα σφαλερὴ, ἡ δὲ κρίσις χαλεπή. Δεῖ δὲ οὐ μὸνον ἑωυτὸν παρέχειν τὰ δέοντα ποιεῦντα, ἀλλὰ καὶ τὸν νοσὲοντα, καὶ τοὺς παρεὸντας, καὶ τὰ ἔξωθεν.*

*Life is short, and the Art long; the opportunity fleeting; the experience perilous, and the decision difficult. The physician must not only be prepared to do what is right by himself, but also to make the patient, the attendants, and externals collaborate *[13]*.*

## Data Availability

No datasets were generated or analysed during the current study.
